# The Youth Aware of Mental Health program in Australian Secondary Schools: 3- and 6-month outcomes

**DOI:** 10.1186/s13033-021-00503-w

**Published:** 2021-10-21

**Authors:** Lauren McGillivray, Fiona Shand, Alison L. Calear, Philip J. Batterham, Demee Rheinberger, Nicola A. Chen, Alexander Burnett, Michelle Torok

**Affiliations:** 1 Black Dog Institute, University of New South Wales, Hospital Road, Randwick, NSW 2031 Australia; 2 Centre for Mental Health Research, Research School of Population Health, Australian National University, Eggleston Road, Acton, ACT 2601 Australia

**Keywords:** Suicide prevention, Suicidal ideation, Adolescence, Youth Aware of Mental Health

## Abstract

**Objective:**

The evidence base for suicide prevention programs in Australian schools is limited. The aim of this study was to examine the impact of a universal, mental health promotion and suicide prevention program—Youth Aware of Mental Health (YAM)—on suicidal ideation, mental health, and help-seeking in Australian secondary school students from baseline to post-intervention and 6-month follow up.

**Methods:**

Using a single-arm design, the YAM program was delivered to Year 9 students (13–16 years) in secondary schools located within four regions across New South Wales, Australia. A structured self-report questionnaire using validated scales was administered at each time point. Linear mixed-effects modelling was used to examine differences in suicidal ideation scores across time, while accounting for random effects of individual schools.

**Results:**

Suicidal ideation reduced significantly from baseline to post, and from baseline to follow-up (*p* < 0.001). Depression severity declined (*p* < 0.001) and help-seeking intentions increased (*p* < 0.001) at post-intervention and 6- months following the intervention period. No suicide deaths were reported for any study participants.

**Conclusion:**

The current findings provide preliminary evidence that the YAM program is a promising preventive intervention for Australian schools, particularly for reducing suicidal ideation, depression and increasing help-seeking intentions in young people. The implementation of YAM in a large number of schools across New South Wales demonstrates the feasibility, and acceptability by schools, of implementing this program at scale.

*Trial registration:* ANZCTR, ACTRN12619000338167. Registered 5 March 2019—Retrospectively registered, http://www.anzctr.org.au/Trial/Registration/TrialReview.aspx?id=376989&isReview=true.

## Background

Young people are recognised in suicide prevention policy as a group needing targeted intervention [37] as youth suicide rates are increasing faster than in adults [6]. In Australia, suicide rates among young people (15–24 years) are at their highest in 10 years, and suicide remains the leading cause of death in this age group, accounting for over one third (38.4%) of total deaths [3]. Moreover, suicidal ideation and attempts far outweigh the number of suicide deaths. Recent nationwide surveys in Australia show that approximately 7.6% [13], 5.2%, and 2.4% of youth (aged 12–17 years) reported suicidal ideation, plans and attempts in the past 12-months, respectively [38, 39]. The heightened risk for suicide in young Australians has spurred a focus on, and considerable investment in, youth mental health and suicide prevention activities [17].

Schools are a logical setting for delivering preventive interventions in childhood and adolescence and should be at the frontline of mental health efforts for reasons of unparalleled reach and access to young people [29–31]. In addition, mental health is inextricably intertwined with academic outcomes [7, 20], such that schools are likely to be motivated to provide meaningful and sustainable wellbeing support for students. Yet, few evidence-based suicide and mental health prevention programs for suicide are being implemented in secondary schools [24], despite this being a developmental period in which suicidal thoughts and behaviours typically emerge [19, 32], and recent meta-analytical evidence that school-based interventions have a preventive effect on suicidal ideation and attempts in youth [22]. Currently, the most robust evidence for suicide prevention is associated with the Youth Aware of Mental Health (YAM) [34] and Signs of Suicide (SOS) [2] programs. These programs are designed to improve early identification and management of young people at risk of suicide through increasing youth awareness and knowledge of mental health, teaching skills in how to respond and seek help for self and peers, and improving coping skills to manage adverse and stressful life events. Randomised controlled trials (RCTs) of SOS have shown reductions in suicide attempts over 3-months [1, 25], while a large-scale RCT of YAM involving 168 schools (*N* = 11,110 students) in 10 European countries has been associated with a 55% reduction in incident suicide attempts and 50% fewer cases of severe suicidal ideation, relative to control, at 12 months post-intervention [34]. Against a background of increasing rates of suicidal ideation and suicide attempts in school-aged youth, it will become increasingly difficult for Australian education departments to meet supply–demand to provide therapeutic support to students with mental health needs within schools. YAM was selected as the school-based program for the current trial due to the strength of the research evaluation design, the comprehensiveness of the program, the enduring effects, and the potential for YAM to be integrated into school practice, thereby helping to manage the increasing mental health burden.

This paper reports on outcome data collected for an evaluation of the YAM program, which was implemented as part of a larger multilevel suicide prevention trial that took place in four regions in New South Wales, Australia [18, 28]. Since YAM was offered to all government, independent and Catholic secondary schools to achieve saturation of the program within each of the trial regions, it was not possible to establish a control condition. Therefore, this is a pilot study that utilised a pre-post design with follow-up. It is important to provide the first Australian evidence for this evidence-based preventive intervention, and if shown to improve suicidal ideation then it provides a promising prevention program for further implementation and wider dissemination in Australian high schools among adolescents aged 13–16 years.

As defined in the protocol paper [18] the aims of the current study were to:

1. Examine change in the primary outcome—severity of suicidal ideation—from baseline to post-intervention and 6-month follow-up;
2. Examine changes in secondary outcomes: incident self-reported suicide attempt, depression severity, help-seeking intentions, help-seeking behaviours, suicide literacy, and suicide stigma, from baseline to post-intervention and 6-month follow-up.

Consistent with the evidence-base for YAM, we hypothesise that suicidal ideation scores will decline over time at post-intervention and 6-month follow-up [34]. Moreover, this age group of young people is in a developmental period when the prevalence of suicidal thoughts and behaviours is typically increasing rapidly [19], so a decline in suicidal ideation would provide compelling evidence for a preventive effect of the intervention. The proportion of new incidents of reported suicide attempt after receiving the YAM program will be exploratory, as well as change in other secondary variables at post-intervention and 6-month follow-up.

## Methods

This study has been reported in line with CONSORT guidelines [27]. Ethics approvals for this study were obtained from the University of New South Wales Human Research Ethics Committee (HC17710),the State Education Research Applications Process committee (2017199), who oversee research conducted in public schools in New South Wales; and the Catholic Education Offices in Maitland-Newcastle, Wollongong, Broken Bay, and Wagga Wagga. This study was registered with the Australian New Zealand Clinical Trials Registry (ACTRN12619000338167) on 05 March 2019. Informed consent was obtained from the school principal of each school, followed by parents/carers of each student, and student assent was collected for participation in the study.

The YAM program was delivered as the schools-based strategy within the LifeSpan trial (ACTRN12617000457347), to Year 9 students (aged 13–16 years) in 76 government (n = 63, 94%) and independent and Catholic (n = 13, 25.5%) secondary schools within the four Lifespan trial regions. YAM was delivered in five classroom sessions over 3 weeks by two trained adults. Youth participate in group discussions and role-plays, of up to 30 students, where they learn about and discuss everyday mental health and are introduced to different methods to improve problem-solving and emotional functioning in difficult real-life situations. In the first session, the instructors work to set a safe environment by introducing mental health using the YAM pedagogical materials that include slides, posters, and a booklet for each participant to keep. These materials include information about the following six topics that also serve as a basis for the discussions and role-plays in YAM: (1) What is mental health, (2) Self-help advice, (3) Stress and crisis, (4) Depression and suicidal thoughts, (5) Helping a friend in need, and (6) Getting advice: who to contact. The booklet includes information about local health and mental health care options as well as youth organisations in their communities. The four sessions that follow differ depending on those present, as the topics to be role-played and discussed are proposed by group members (for more detail of the program see [33]. Although 76 schools received the YAM program, not all schools nor all students were evaluated due to either lack of school response to the study invitation, a recent critical incident within the school (e.g., death by suicide or suicide attempt of a student), or lack of parent/carer consent. A total of 18 schools (23.7%) agreed to participate in the evaluation and of the total pool of 2,858 students in these schools, 19.4% (*N* = 556) had parental consent to participate in the research and completed the baseline assessment. As YAM is a universal intervention there were no restrictions on individual student participation and thus individual screening for eligibility was not necessary. The YAM program was delivered in a staggered approach, consistent with the broader LifeSpan stepped wedge design, between September 2017 and March 2020. Data were collected at three time-points: pre-intervention (baseline), 3-months post-intervention, and then at 6 months post-intervention follow-up. A fourth, 12-month follow-up time-point was abandoned due to the coronavirus (COVID-19) pandemic affecting school closures.

## Measures

### Suicidal ideation (primary outcome) and suicide attempt

Paykel’s Suicidal Feelings in the General Population Questionnaire [21] was used to measure suicidal ideation. This questionnaire includes four items and asks respondents to rate their experience of suicidal ideation and intent in the previous two weeks from 1 (never) to 6 (always). A total score of 4–24 can be calculated, with higher scores indicating a higher degree of suicidal ideation. A total score above four was used to indicate the presence of suicidal ideation, which was converted into a dummy variable (yes/no) for reporting on prevalence of suicidal ideation only. This scale demonstrated excellent internal consistency (α = 0.94) in the present sample.

A fifth item in the Paykel’s Suicidal Feelings in the General Population Questionnaire was used to assess history of suicide attempt, where the respondent answers Yes/No to the question, ‘Have you ever tried to take your own life?’. An additional item was added to further assess the timeframe of previous suicide attempts, asking ‘When did you try to take your own life?’, with response options of’during the past two weeks’, ‘during the past year’, or ‘earlier’.

### Demographics

To describe the sample, the following demographic characteristics were measured: age, sex, and sexual orientation.

### Depressive symptoms

Depressive symptoms were measured by the Patient Health Questionnaire Depression Scale (PHQ-8) [16]. This scale consists of 8 items that cover the DSM-IV symptoms of depression. Items are rated on a 4-point scale, ranging from 0 (not at all) to 3 (nearly every day), to indicate how often the participant has been experiencing each symptom during the past two weeks. For example, “Have you felt down, depressed, or hopeless?”. Higher scores indicate the presence of more depressive symptoms, and the maximum total score is 24. This scale demonstrated good internal consistency (α = 0.89) in the present sample.

### Help-seeking intention

The General Help-Seeking Questionnaire (GHSQ adapted) was developed in Australia [11, 36] and assesses intentions to seek help for personal or emotional problems from 11 different formal (e.g., psychologist) and informal (e.g., friend) sources. Respondents indicate how likely they are to seek help from each of the sources on a scale ranging from 1 (extremely unlikely) to 7 (extremely likely), with higher total scores indicating greater intentions to seek help for a personal or emotional problem. The overall measure has been shown to demonstrate good to excellent validity and reliability (α = 0.85, test–retest reliability assessed over a 3-week period = 0.92) in Australian high school students [36], and acceptable internal consistency (α = 0.76) in the present sample.

### Help-seeking behaviour

The Actual Help-Seeking Questionnaire (AHSQ adapted [23, 36]), assesses recent help-seeking behaviour in which the respondent indicates whether they have or have not (yes/no) sought help for a mental health problem from 11 different formal (e.g., psychologist) and informal (e.g., friend) sources in the past 3 months. Scores are added to determine the total number of help sources sought (ranging from 0 to 11), with higher total scores indicating greater help-seeking behaviour. This scale demonstrated good internal reliability (α = 0.80) in the present sample.

### Suicide literacy

Suicide literacy was measured with the Literacy of Suicide Scale (LOSS)—short form [8]. The LOSS consists of 12 statements assessing knowledge of suicide where respondents rate each statement based on whether they think it is “True”, “False” or “Don’t know”. The scale provides a total literacy score (ranging from 0 to 12), with higher scores indicating greater suicide literacy/knowledge.

### Suicide stigma

Suicide Stigma was measured with the Stigma of Suicide Scale (SOSS)—short form [5]. The SOSS has 16 items that load onto three subscales: stigma (8 items), isolation/depression (4 items), and glorification/normalisation (4 items). Participants are asked the extent to which they agree with each descriptor of a person who dies by suicide on a 5-point Likert scale ranging from 1 (strongly disagree) to 5 (strongly agree). Higher scores on the stigma subscale (out of 40) are suggestive of greater suicide stigma. The overall short form scale has demonstrated acceptable reliability (Cronbach's alpha = 0.70) and validity for assessing attitudes to suicide [5]. Moderate correlation (*r* = 0.46, p < 0.001) between the LOSS and SOSS indicates high convergent validity, yet also suggests discriminant validity, suggesting that the two scales are assessing separate constructs [4]. The SOSS-stigma subscale demonstrated good internal reliability (α = 0.89) in the present sample.

### Statistical analysis

#### Power analyses

Power calculations were conducted on the assumption that average class sizes would be 25 students, with a total of 1000 students from 20 schools (5 per region) if at least two year 9 classes per school were recruited. Based on a previous school-based trial [9], a non-participation rate of 30% was expected, resulting in data being available from 700 students. Based on repeated measures analysis examining change within factors, a Cohen’s *d* = 0.19 [35] with 80% power and a correlation of 0.5 between measures would require a total sample of *n* = 180. After adjusting for a more conservative design effect of 1.64 (corresponding to an ICC of 0.08) and attrition of 30%, we required a sample of *n* = 421 to meet the research aims, allowing for statistically significant results related to identifying change in suicidal ideation across time.

#### Attrition

Correlates of attrition were identified using *χ*^*2*^ tests for categorical variables and *F* tests from one-way analysis of variance (ANOVA) for continuous variables, comparing participants who did not complete all assessments (*N* = 556) with those who did (*n* = 203).

#### Outcome analyses

Alpha was set at *p* < 0.05 for interpreting significant effects. The following guidelines were used to interpret effect sizes [10]: correlation coefficient (*r*) values of 0.10, 0.30, 0.50, and 0.70, correspond to small, medium, large, and very large effect sizes, respectively. All data were analysed using SPSS version 25.0. The primary analysis for all the outcomes reported here was an intention-to-treat analysis, which included all participants (*N* = 556).

To address Aim 1, linear mixed-effects modelling (LMM) was used to test for intervention effects over time on severity of suicidal ideation, with a repeated effect of time to account for change, and a random effect of school to account for clustering of students within schools. In step one, there was a fixed effect of time. The benefit of mixed-effects models is that they produce unbiased estimates even when some individuals have missing observations, adjust for differential loss to follow-up, and account for clustering by schools and individuals, as required for our data.

To address Aim 2, descriptive analysis via frequency counts were used to determine incident and repeated suicide attempts at post and follow-up time-points. LMMs were used to test for intervention effects over time on secondary outcomes (depression, help-seeking intention, help-seeking behaviour, suicide literacy, and suicide stigma), with repeated effect of time, and random effect of school.

## Results

Among the 556 students, the mean age was 14.4 years (range: 13–16, *SD* = 0.56). Just over half the students identified their sex as female (56.3%) and their sexual orientation as heterosexual (91%).

### Attrition

Of the 556 participants who completed baseline surveys, 327 completed the 3-month post survey and 327 completed the 6-month follow-up survey. Only 203 participants completed surveys at all three timepoints. Attrition was significantly associated with survey mode, where participants completing surveys online (compared to paper) were more likely to complete all follow-up surveys. ANOVA and Chi-Square tests showed no other significant differences in baseline study characteristics between groups (Table 1).

**Table 1:** Baseline sample characteristics for participants with partial completion (N = 556) vs full completion (n = 203)

	*N* = 556	*N* = 203	χ^2^ (*p)*
	Count (%)	Count (%)	
Survey mode			
Paper	313 (56.3)	79 (38.9)	17.98 **(0.000)**
Online	243 (43.7)	124 (61.1)	
Sex			
Male	240 (43.4)	86 (42.8)	0.02 (0.880)
Female	313 (56.6)	115 (57.2)	
Sexual orientation			
Heterosexual LGBQ + ^a^	506 (92.3) 42 (7.7)	184 (91.1) 18 (8.9)	0.31 (0.577)
Suicide attempt			
Yes	42 (7.6)	18 (9.0)	0.37 (0.543)
No	508 (92.4)	182 (91.0)	

	Mean (*SD*)	Mean (*SD*)	*F (p)*
Suicidal ideation	6.59 (4.19)	6.47 (4.39)	0.12 (0.729)
Depression	6.91 (5.87)	7.21 (6.21)	0.37 (0.541)
Help-seeking intention	38.07 (11.12)	37.73 (10.15)	0.14 (0.706)
Help-seeking behaviour	0.57 (1.38)	0.52 (1.25)	0.21 (0.644)
Suicide literacy	6.15 (2.32)	6.43 (2.31)	2.16 (0.142)
Suicide stigma	29.04 (7.08)	29.06 (6.99)	0.01 (0.928)

Bold values indicate *p* < 0.05

^a^LGBQ + refers to Lesbian, Gay, Bisexual, Queer

### Change in suicidal ideation across time

A total of 552 (of 556) participants answered the suicidal ideation assessment at baseline. The proportion of participants reporting the presence of suicidal ideation (total score > 4) decreased from baseline (*n* = 285, 51.6%) to 3-month post-intervention (*n* = 125, 38.6%) and 6-month follow-up (*n* = 130, 39.9%). Of the 552 who had a baseline suicidal ideation measure, 199 had a paired 6-month measure so we were able to determine the proportion of participants that reported remission of suicidal ideation at 6-month follow-up, which was 21.6% (*n* = 43). Table 2 presents fixed effects output from the LMM. Change in suicidal ideation scores over time was significant, *F*(2, 329.36) = 8.45, *p* < 0.001. Mean suicidal ideation score reduced significantly from baseline (*M* = 6.60, *SD* = 4.19) to 3-month post-intervention (*M* = 5.69, *SD* = 3.17), *p* < 0.001, *d* = 0.22, and from baseline to follow-up (*M* = 5.87, *SD* = 3.44), *p* < 0.001, *d* = 0.17, but not from 3-month post-intervention to 6-month follow-up.

**Table 2:** Mixed-models estimates of fixed effects for mean change in suicidal ideation over time (N = 556)

Predictor/effect	Estimate	SE	df	*t (p)*	95% CI
Time (post)	− 0.60	0.16	417.63	− 3.76 (**0.000**)	− 0.92 to − 0.29
Time (follow-up)	− 0.65	0.18	436.64	− 3.59 (**0.000**)	1.00 to − 0.29

Bold values indicate *p* < 0.05; Time (baseline) is the reference category

### New incidents of suicide attempt and change in secondary outcomes across time

In total, 7.6% (n = 42/550) of participants reported a lifetime suicide attempt at baseline, 8.7% (n = 28/321) at 3-month post-intervention, and 7.8% (n = 25/322) at 6-month follow-up. There were 13 new cases of suicide attempt among participants who had not reported an attempt at baseline, with eight of these reported at 3-month post-intervention and eight reported at 6-month follow-up. There were no new cases reported at 6-month follow-up relative to 3-month post-intervention. Of the 13 new cases, five were reported to have occurred earlier than 12-months ago, meaning they would have been valid at baseline. Therefore it is unlikely that these five attempts represent incident suicide attempts, and more likely that they were not reported accurately when first asked, at baseline. If these five reports were included, the baseline rate of lifetime suicide attempt in this sample would increase from 7.6% (n = 42/550) to 8.5% (n = 47). In addition, there were nine participants who responded ‘yes’ to the question about lifetime history of suicide attempt at baseline and ‘no’ when asked this same question later. Given this inconsistency, the baseline rate of lifetime history of suicide attempts is between 6.0% and 8.5%. No suicide deaths were reported for any study participant.

Table 3 presents fixed effects output from the LMM testing change in secondary outcomes over time. Change in depression scores over time was significant, *F*(2, 355.82) = 10.97, *p* < 0.001, with significant reductions from baseline (*M* = 6.91, *SD* = 5.87) to 3-month post-intervention (*M* = 5.85, *SD* = 5.44), *p* < 0.001, *d* = 0.18, and from baseline to 6-month follow-up (*M* = 6.04, *SD* = 5.68), *p* < 0.001, *d* = 0.15, but not from 3-month post-intervention to 6-month follow-up. There was significant change in help-seeking intentions over time, *F*(2, 377.82) = 13.18, *p* < 0.001, with significant increases from baseline (*M* = 38.07, *SD* = 11.12) to 3-month post-intervention (*M* = 39.95, *SD* = 10.24), *p* < 0.001, *d* = 0.17, and from baseline to 6-month follow-up (*M* = 40.56, *SD* = 11.21), *p* < 0.001, *d* = 0.22, but not from 3-month post-intervention to 6-month follow-up. Help-seeking behaviour (*M* = 3.01, *SD* = 1.67; *M* = 2.98, *SD* = 1.44; *M* = 3.19, *SD* = 1.82), suicide literacy (*M* = 6.15, *SD* = 2.32; *M* = 6.60, *SD* = 2.44; *M* = 6.18, *SD* = 2.74), and suicide stigma (*M* = 29.04, *SD* = 6.98; *M* = 29.50, *SD* = 6.95; *M* = 29.38, *SD* = 6.51) scores did not change significantly from baseline to 3-month post-intervention nor 6-month follow-up, respectively.

**Table 3:** Mixed-models estimates of fixed effects for mean change in secondary variables over time (N = 556)

Predictor/effect	Estimate	SE	df	*t (p)*	95% CI
Depression					
Time (post) Time (follow-up)	− 1.05 − 0.92	0.24 0.26	385.30 386.39	− 4.28 (0**.000**) − 3.49 (0**.000**)	− 1.53 to − 0.57 − 1.43 to − 1.42
Help-seeking intention					
Time (post) Time (follow-up)	1.97 2.65	0.51 0.56	380.78 392.39	3.85 (**0.000**) 4.76 (0**.000**)	0.96 to 2.97 1.56 to 3.75
Help-seeking behaviour					
Time (post) Time (follow-up)	0.03 0.02	0.06 0.07	358.53 379.88	0.52 (0.605) 0.23 (0.815)	− 0.09 to 0.16 − 0.12 to 0.16
Suicide literacy					
Time (post) Time (follow-up)	0.22 0.02	0.12 0.13	372.91 367.60	1.88 (0.060) 0.14 (0.889)	− 0.01 to 0.45 − 0.24 to 0.28
Suicide stigma					
Time (post) Time (follow-up)	− 0.00 0.59	0.36 0.34	391.07 395.80	− 0.01 (0.992) 1.74 (0.083)	− 0.71 to 0.70 − 0.08 to 1.25

Bold values indicate *p* < 0.05; Time (baseline) is the reference category

## Discussion

The current paper provides the first Australian evidence of the impact of Youth Aware of Mental Health (YAM) as a potential intervention to reduce suicidal thoughts and behaviours in Australian young people. This pre-post pilot study found that YAM participation was associated with a reduction in the severity of suicidal ideation at 3-month post-intervention and 6-months follow-up, supporting the first hypothesis. This aligns with previous findings from a large-scale RCT [34], which showed that the YAM program reduced the incidence of severe suicidal ideation, and suicide attempts, among adolescents in different counties across the European Union. Moreover, the present findings showed similar effects, with more than one-fifth of students who reported suicidal thoughts before the program started going on to report no suicidal thoughts at 6-months after receiving the program. Though we had no control group, this is compelling evidence of change in a naturalistic setting where the prevalence of suicidal ideation is typically increasing rapidly in this age group [19].

Thirteen participants (4.0%) reported an incident suicide attempt occurring after the baseline survey was collected. This is much higher than what would be expected over this time period, and following a suicide prevention intervention (e.g., 0.7% incident suicide attempts at 12-months following YAM [34]. However, given that five of these cases were reported to have occurred earlier than 12-months ago (overlapping with the baseline timepoint), it means that they were either inaccurate recollections of an attempt—similar to what has been reported in other adolescent studies (see [12, 15])—or the baseline rate of suicide attempt in the current sample was greater than originally recorded. The range of suicide attempts reported in this participant sample (6.0%—8.5%) is consistent with previous research reporting rates between 6.1% [26] and 11.7% [14] in secondary school-aged youth.

YAM participation was associated with a reduction in depression severity and with an increase in help-seeking intentions at 3-month post-intervention and 6-month follow-up, which persists for at least 6-months. Increased help-seeking intentions in the current sample will likely provide opportunities to link these youth to appropriate mental health professionals and services that can offer targeted support for suicidal ideation and depression.

### Implications

There are several implications of the current findings for schools and future researchers. The implementation of the YAM program in a large number of Australian schools across New South Wales demonstrates the feasibility of implementing this program at scale, as well as the acceptability of the program given the high number of schools signing-on to receive the program. Schools are well positioned to embed mental health and suicide prevention programs into current wellbeing initiatives and school policy, with the sustainment of evidence-based interventions being a priority. YAM has the potential to help manage the increasing mental health burden for schools, through reducing suicidal ideation and depression severity, and enhancing intentions of youth to seek help before a mental disorder becomes established (and more burdensome to treat). To build on the current research, what is now needed are large-scale hybrid effectiveness-implementation RCTs that would not only definitively establish that YAM reduces suicidal ideation and attempts in Australian youth, but could also establish the conditions needed for it to be integrated into routine teaching practices of schools to ensure all youth are exposed to the program. This could also involve examination of the collateral impact of this program on educators and other professional staff working at schools that deliver YAM.

### Limitations

A number of limitations of the current study need to be acknowledged. First, there was no control or comparative group, rather the study was based on cross-sectional self-report data, and thus we cannot definitively attribute treatment effects to the YAM program. However, given that students who received YAM are in a developmental period when the prevalence of suicidal thoughts and behaviours is typically increasing rapidly, the marked decline in suicidal thoughts in the present study provides compelling evidence for a preventive effect of the YAM program. Second, while statistically significant, it is not clear that changes in suicidal ideation scores would equate to an observable benefit that students would notice as an improvement in their emotional wellbeing or functioning. Third, fewer than one quarter of eligible schools agreed to participate in the evaluation of YAM. If all schools had signed on, we would have had more robust data to ascertain the preventive effects at scale. It is only by having schools participate in research evaluation that we are able to establish whether interventions such as YAM are making a measurable difference to the students they are intended to benefit. Fourth, the high attrition rate for participants completing paper-based surveys may have impacted the representativeness of the study sample. Schools with less access to technological resources may have been more likely to opt for paper-based surveys, which could bias the sample towards schools with greater socioeconomic advantage. Fifth, the incidence of suicide attempts could not be reliably assessed due to inconsistent reporting. Participants who reported a history of suicide attempts on baseline surveys were contacted by their school counsellor/psychologist, and some may have wanted to avoid disclosing this again in subsequent surveys. Alternatively, some participants may have changed their perception of whether their self-harm behaviour was considered a suicide attempt or not, or simply forgot [15].

## Conclusions

The evaluation of YAM, as part of the LifeSpan research trial, has contributed to the presently scarce evidence base for universal suicide prevention programs in Australian schools. Overall, the current findings provide preliminary support that the YAM program is a promising preventive intervention for schools, particularly for reducing suicidal ideation and depression, and increasing help-seeking intentions in young people. The comprehensiveness of the YAM program, its enduring effects, and the potential for YAM to be integrated into school practice makes it a valuable intervention to help manage the increasing mental health burden in Australian young people. This study also shows that the YAM program appears to be acceptable to schools given the high number of school signing-on to receive the program.

## Data Availability

The datasets generated and analysed for the current study are not publicly available due to sensitive nature of suicide-related data and age of the sample (< 18 years).

## Abbreviations

YAM: Youth Aware of Mental Health
SOS: Signs of Suicide
RCT: Randomised controlled trial
PHQ-8: Patient Health Questionnaire Depression Scale
GHSQ: The General Help-Seeking Questionnaire
AHSQ: The Actual Help-Seeking Questionnaire
LOSS: Literacy of Suicide Scale—short form
SOSS: Stigma of Suicide Scale—short form
ANOVA: Analysis of variance
LMM: Linear mixed-effects modelling
